# The Impact of Beta-Catenin and glutathione-S-transferase Gene Polymorphisms on the Treatment Results and Survival of Multiple Myeloma Patients

**DOI:** 10.1007/s12253-019-00747-5

**Published:** 2019-09-10

**Authors:** Ildikó Pál, Bernadett Szilágyi, Béla Nagy, Tibor Pál, Katalin Hodosi, Árpád Illés, László Váróczy

**Affiliations:** 1 Department of Hematology, Faculty of Medicine, University of Debrecen, Nagyerdei krt. 98., Debrecen, H-4032 Hungary; 2 Department of Laboratory Medicine, Faculty of Medicine, University of Debrecen, Debrecen, Hungary

**Keywords:** Multiple myeloma, Cereblon, ß-catenin, Glutathione-S-transferase, Polymorphism, Personalized treatment

## Abstract

Multiple myeloma (MM) is an incurable disease, however, novel therapeutic agents has significantly improved its prognosis. In this study we analyzed if polymorphisms in the genes of β-catenin and glutathione-S-transferase have affected the clinical course, treatment response and progression-free survival (PFS) of MM patients. Ninety-seven MM patients were involved who were administered immunomodulatory drug (Imid) or alkylating agent-based therapy. *β-catenin* (*CTNNB1*, rs4135385 A > G, rs4533622 A > C) and *glutathione-S-transferase* (GSTP1 105, GSTP1 114) gene polymorphisms were analyzed by Light SNiP assays. The distribution of *CTNNB1* (rs4135385) AA, AG and GG genotypes were 48.4%, 47.4% and 4,1%, respectively. Patients with AA genotype were older than those who carried G allele (64.5 vs. 61.0 years of age, *p* < 0.05). Response to Imid-based therapies (p < 0.05) and PFS (*p* = 0.032) were significantly more favourable in the AA homozygous group. The other polymorphism (rs4533622) of β-*catenin* gene did not markedly influence these clinical parameters, although MM was diagnosed at significantly younger age in subjects with CC genotype compared to AG/AA combined genotypes (59.1 vs. 65.7 years, *p* = 0.015). When *GSTP1* polymorphisms were investigated, no such significant associations were observed. Our results demonstrate that the polymorphism of *β-catenin* gene (rs4135385) may be an independent predictive factor in MM.

## Introduction

Multiple myeloma (MM) is a malignant hematologic disorder that accounts for 1 % of all cancer cases. It is considered as an incurable disease however there has been a significant improvement in patients’ survival, thanks to the novel drugs that have been recently introduced. Subclonal heterogeneity of MM has been described by several studies. The heterogenous molecular pattern of MM results in different therapeutic responses [1]. Due to molecular heterogeneity, there is a need to identify new biomarkers of drug resistance. Biomarker tests can improve the efficacy of traditional therapies, therefore determination of molecular profile in cancer patients is becoming standard of care [2]. In multiple myeloma, new therapies include immunomodulatory agents (IMIDs), proteasome inhibitors (PI) and monoclonal antibodies [3]. Thalidomide, lenalidomide and pomalidomide are immunomodulatory drugs that can be administered in first-line treatment, maintenance and/or relapse settings of MM. Imids act via the cereblon-β-catenin pathway. Cereblon (*CRBN*) is expressed in several malignant cell lines, such as myeloma and lymphoma cells. It is a part of the CRL4 complex that has a major role in the ubiquitination of IKZF1 and IKZF3 transcription factors [4, 5]. Thalidomide binds to cereblon and inhibits its E3 ubiquitin ligase function. Moreover, thalidomide facilitates upregulation of p21 and downregulation of interleukine-8, that results in an arrest in the G0/G1 transition of the cell cycle [6, 7]. However, myeloma cells are able to become resistant against Imids which process is still not clear in details. Lately, the pathological regulation of β-catenin/Wnt pathway has been described in the lenalidomide resistance setting. β-catenin plays a key role in the Wnt nuclear transcription pathway. It is phosphorylated by the Axin/CK1a/APC/GSK3 complex, then it is transported to the nucleus, which results in an improvement of the survival rate of tumor cells. Lenalidomide and thalidomide increase the intracellular concentration of β-catenin, but also facilitate the expression of c-myc and other anti-apoptotic factors, and treatment may become ineffective consequently. Overall, cereblon is responsible for the acitivity of Imids – thalidomide and lenalidomide -, but the Wnt/β-catenin pathway may contribute to the development of resistance against these drugs [8–10]. Genes that encode cereblon (*CRBN*) and β-catenin (*CTNBB1*) can be found on chromosome 3 in humans. The exchange of adenine and guanine in intron 13 is responsible for rs4135385 polymorphism of *CTNBB1* gene, while rs4533622 polymorphism incorporates an adenine-citozine substitution [4, 5]. *CTNBB1* (rs4533622) polymorphism was reported to play a role in the clinical course of MM, on the other hand, rs4135385 polymorphism can be a predisposing factor at the onset of the disease. Cyclophosphamide – thalidomide – dexamethasone (CTD) therapy was found to be more effective and less progression was observed in *CTNBB1* (rs4135385) AA homozygous patients. Patients carrying the *CTNNB1* (rs4533622) AA genotype were better responders to the first-line therapy with thalidomide-containing regimens. These results may highlight the need for administering personalized treatment for MM patients [11, 12].

The polymorphisms of some other drug metabolizing enzyme genes may also influence treatment efficacy in MM patients. For instance, glutathione-S-transferase (GST) enzymes are responsible for the detoxification of several xenobiotics and chemotherapeutical agents [13]. *GSTP1* enzyme is an important member of the GST family, as it metabolizes melphalan, cyclophosphamide, vincristine, doxorubicine, cisplatina, etoposide and chlorambucil. Environmental carcinogens, such as benzene, dioxins, and fume are also the substrates of *GSTP1* [14]. Aminoacid substitutions of Ile105Val and Ala114Val are likely to have a significance in the modulation of enzyme activity [15]. In patients having *GSTP1* 105 variant genotype, detoxification process was less active, therefore chemotherapeutical agents can be more effective. In MM patients carrying *GSTP1* 105Ile homozygous genes, progression-free (PFS) and overall survival results were reported to be more favourable [16].

Our aim was to investigate if *CRBN*, *CTNNB1* and *GSPT1* gene polymorphisms have any effect on treatment response and survival data of a large group of Hungarian MM patients and if administration of personalized treatment can be considered in this disease.

## Patients and Methods

### Clinical Data

The clinical files of multiple myeloma patients were reviewed with particular reference to age, sex, clinical stage, response to treatment and survival. ISS stages were determined using the International Myeloma Working Group criteria. FISH results of unfavourable prognosis included t(4;14), t(14;16) and del(17p). Examining the survival rates, overall survival (OS) was determined by consideration of death events due to any reasons, while progression-free survival (PFS) was determined by consideration of relapses, deaths or disease progression that indicated further treatment. Descriptive statistical analysis was used to characterize the patient populations. Normality of the parameters were examined applying the Wilk-Saphiro test. Comparing two groups, F probe and t test were administered by normal distribution of the parameters, otherwise the non-parametrical Mann-Whitney test was applied. Differences were significant if probability level was less than 5% (*p* < 0.05). Survival rates were calculated using the Kaplan-Meier’s method, while the survival data were compared using the log-rank test. Cox uni- and multivariable regression analysis were used to assess several risk factors simultaneously in terms of survival data.

### Determination of Gene Polymorphisms

#### Genomic DNA Extraction

DNA for genotyping was extracted from peripheral blood samples obtained into K_3_-EDTA Vacutainer tubes (Becton Dickinson, San Jose, CA, USA) using QiaAmp DNA Blood Mini Kit (Qiagen GmbH, Germany) according to the manufacturer’s recommendations.

#### Genotyping of the CRBN, CTNNB1 and GSTP1 Gene Polymorphisms

LightSNip typing assays were applied to determine *CRBN* (rs121918368C > T), *CTNNB1* polymorphisms (rs4135385 A > G, rs4533622 A > C) and *GSTP1*105 (rs1695A > G) and 114 (rs1138272C > T) (TIB-MolBiol, Berlin, Germany) on a LightCycler 480 Real-time PCR Instrument (Roche Diagnostics, Mannheim, Germany). Amplifications were performed based on the recommendations of the manufacturer. PCR reaction was carried out in 20 μl volume containing the LightCyclerFastStart DNA Master HybProbe (Roche Diagnostics). Samples were run in duplicates.

## Results

Ninety-seven multiple myeloma patients were involved in the study who were treated in our institution between January 2012 and December 2016. Their mean age was 62.47 years at diagnosis, male-female ratio was 43:54. The distribution of ISS1, 2 and 3 stages were 27.8%, 42.3% and 29.9%, respectively. FISH tests were performed in 67 patients and 42 (62.7%) of them had a result of unfavourable prognosis. Treatment modalities included thalidomide-based (59 cases), lenalidomide-based (18 cases), and alkylating-agent based (57 cases) regimens. Thirty-eight patients received two or more treatment lines.

In this cohort, the distribution of *CTNNB1* (rs4135385) AA, AG and GG genotypes were 48.5%, 47.4% and 4.1%, respectively. Patients with AA genotype were older than those who carried G allele (64.5 vs. 61.0 years of age, *p* = 0.05). However, the presence of the *CTNNB1* (rs4135385) AA genotype did not show association with ISS stage or FISH test results (Table 1). Regarding the rs4533622 polymorphism of the *CTNNB1*, the different genotypes were detected in the following ratio: AA genotype in 18.5%, AC in 50.5% and CC in 31% of these patients. MM was diagnosed at significantly younger age in those carrying the CC genotype (59.1 vs. 65.7 years, *p* = 0.015). Additionally, females were more likely to bear the AA genotype (Table 2). Similarly to the other polymorphism of β-catenin, there was no relationship between allele distribution and clinical stages or FISH results. In terms of the *CRBN* (rs121918368) polymorphism, all patients carried the CC genotype, thus, no further analysis was performed in regard to clinical features, treatment results and survival ratio.

**Table 1:** *CTNNB1* (rs4135385) polymorphism and its relationships with demographical and other clinical parameters

	AA	G carrier	p
Number of patients (%)	48 (49.5)	49 (50.5)	
Male: female ratio	18:30	25:24	0.180
Median age at diagnosis (years)	64.5	61.0	**0.040**
ISS stage			
I	11	16	0.406
II	20	21	
III	17	12	
FISH result			
standard	13	12	0.592
unfavourable	19	23	
Number of cases receiving Imid-based therapy	28	48	
Response			
CR + VGPR	16	21	**0.040**
PR	11	15	
NR	1	12	
Imid-related side effects			
polyneuropathy	4	4	0.72
neutropenia	1	8	1.0
anaemia	1	7	1.0

**Table 2:** *CTNNB1 (rs4533622)* polymorphism and its relationships with demographical and other clinical parameters

	AA	AC	CC	p
Number of patients (%)	18 (18.5)	49 (50.5)	30 (31)	
Male: female ratio	2:16	27:22	14:16	**0.005**
Median age at diagnosis (years)	68	64	59	**0.015**
ISS stage				
I	4	16	7	0.417
II	6	19	16	
III	8	22	7	
FISH result				
standard	5	10	10	0.479
unfavourable	9	22	11	
Number of cases receiving Imid-based therapy	11	36	29	
Response				
CR + VGPR	5	21	7	0.438
PR	5	9	4	
NR	1	6	3	
Imid-related side effects				
polyneuropathy	1	2	5	0.22
neutropenia	1	3	5	0.88
anaemia	1	3	4	0.93

For the GSTP1 105 polymorphism, there were 54 (55.7%) patients carried AA (Ile/Ile), 33 (34%) had AG (Ile/Val) and 10 (10.3%) had GG (Val/Val) genotype. When GSTP1 114 polymorphism was tested among these MM individuals, CC (Ala/Ala) genotype was found in 85 (87.6%) subjects, while CT (Ala/Val) genotype was found in 12 (12.4%) patients. No association was observed between these polymorphic variants in *GSTP1* and demographical data.

**Fig. 1:**
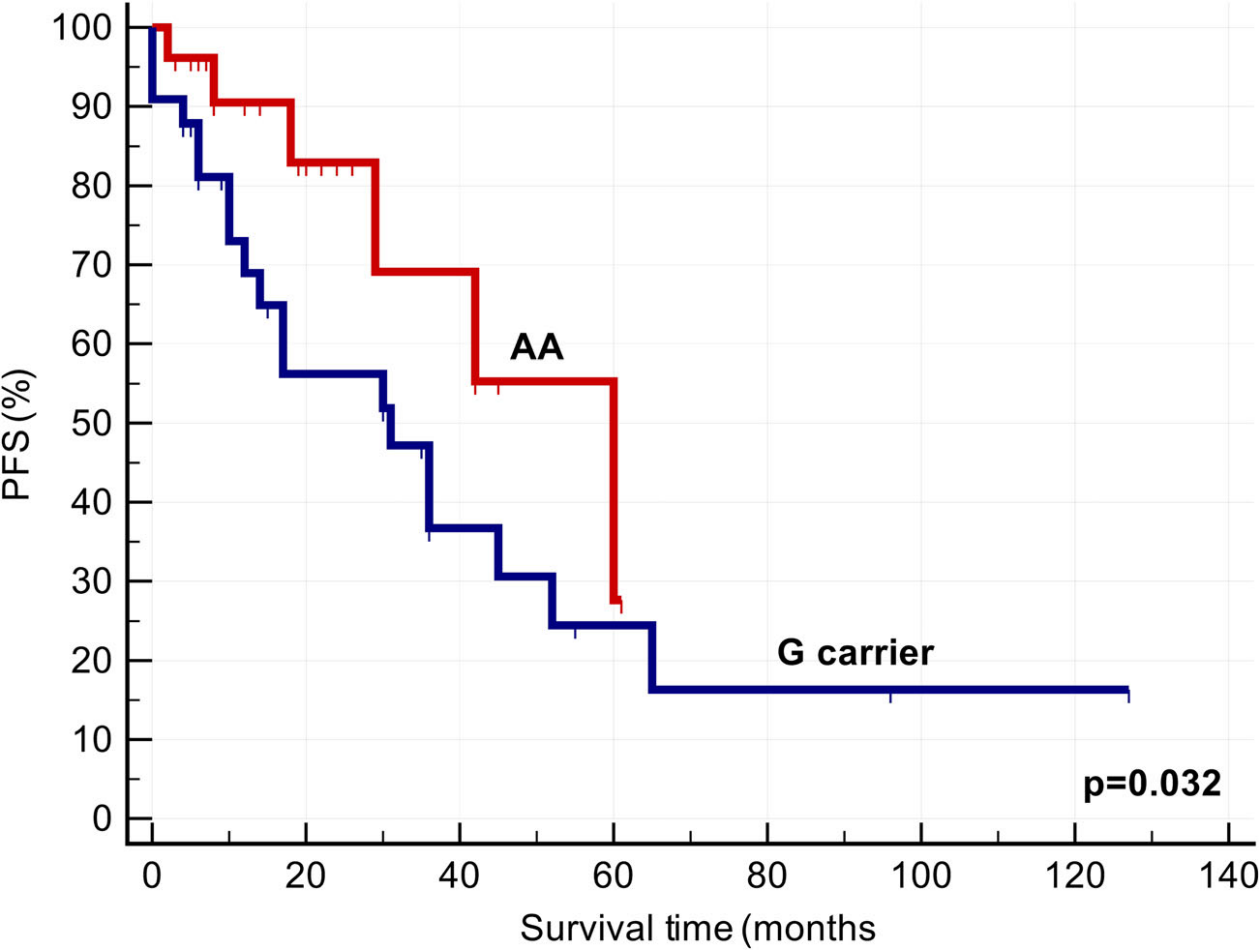
Progression-free survival after Imid-based therapies in case of CTNNB1 (rs4135385) polymorphisms

**Fig. 2:**
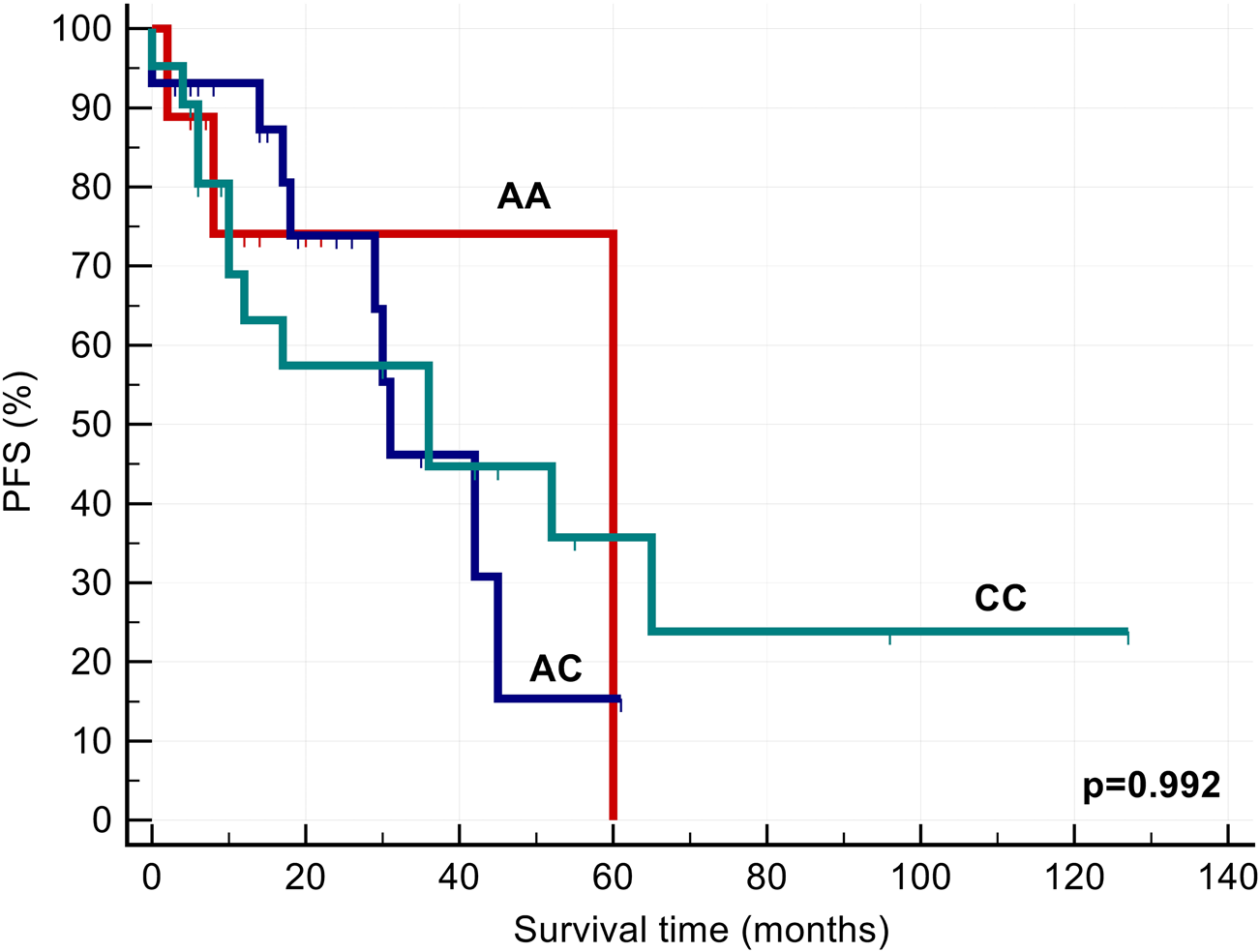
Progression-free survival after Imid- based therapies in case of CTNNB1 (rs4533622) polymorphisms

We also investigated the effect of *CTNNB1, CRBN and GSTP1 polymorphisms* on response to chemotherapy. We found that response to Imids-based therapies (*p* < 0.05) and PFS (*p* = 0.032) were significantly more favourable in AA homozygous group (Table 1, Fig. 1). Cox univariable (HR: 2.371 [1.026–5.477], *P* = 0.043), and multivariable analysis tests (HR: 2.554 [1.099–5.933], *P* = 0.029) for PFS proved that *CTNNB1* (rs4135385) polymorphism can be considered as an independent prognostic factor besides ISS stages and FISH results (Tables 3 and 4). In patients with stage II and stage III disease, G carriers were expected to have more unfavourable survival results than AA homozygous individuals (Table 5). In contrast, the other polymorphism (rs4533622) of β-catenin gene did not markedly influence the effectivity of thalidomide and lenalidomide-based therapies (Table 1). Progression-free survival was markedly favourable in the AA group; however, the survival curves did not differ significantly from each other (Fig. 2). β-catenin gene polymorphisms did not have any impact on the occurrence of common side effects of the Imid-based therapies (Tables 1 and 2).

**Table 3:** Univariable Cox regression analysis for PFS in terms of ISS stage, FISH results and *CTNNB1* (rs4135385) polymorphism

	HR (95% CI)	p
ISS stage II vs I	1.177 (0.541–2.696)	0.701
III vs I	2.780 (1.056–7.321)	0.038
FISH result unfavourable	2.927 (1.088–7.872)	0.033
rs4533622	2.371 (1.026–5.477)	0.043
G carrier vs AA

**Table 4:** Multivariable Cox regression analysis for PFS in terms of ISS stage and *CTNNB1* (rs4135385) polymorphism

	HR (95% CI)	p
ISS stage II vs I	1.239 (0.540–2.839)	0.513
III vs I	3.179 (1.176–8.590)	0.023
rs4533622	2.554 (1.099–5.933)	0.029
G carrier vs AA

**Table 5:** Estimated PFS in different ISS stages in terms *CTNNB1* (rs4135385) polymorphism

	Estimated survival time (mean, 95% CI) (months)	
	G carrier	AA
ISS I	50.5 (17.4–83.6)	40.2 (18.6–61.8)
ISS II	30.1 (18.0–42.2)	49.5 (35.1–63.9)
ISS III	7.4 (2.2–12.5)	26.1 (6.6–45.6)

Interestingly, when GSTP1 105 and 114 polymorphisms were investigated, no significant association was observed between the response to chemotherapy or survival ratio of these MM patients.

## Discussion

Recently, it has been becoming a trend to administer personalized treatment modalities in cancer patients to improve survival data. Molecular profiling can be a good tool for effective personalized care [2]. In our study, the gene polymorphisms of the cereblon-beta-catenin pathway and glutathione-S-tranferase were investigated in multiple myeloma patients. Several publications have highlighted the importance of *CRBN* gene expression in the effectivity of Imid-based therapies. Thalidomide resistance may occur in case of a low *CRBN* activity, while high levels of *CRBN* mRNA were found to be associated with better responses to thalidomide therapy [17, 18]. Our patient population was homogenous in terms of the *CRBN* gene polymorphism as each of them carried the CC genotype. On the other hand, we could find some links with the *CTNNB1* (rs4533622) polymorphism. In CC homozygous patients, the onset of the disease occurred at a significantly younger age and A alleles were significantly less common in males. These associations have never been published as yet. *CTNNB1* (rs4533622) polymorphism was reported to influence the ISS stages as patients carrying an A allele had more advanced disease. Butrym et al. also described better treatment responses and more neutropenic events in AA homozygous patients [11]. However, we could not confirm such associations in our cohort.

A few studies investigated the role of *CTNNB1* (rs4135385) polymorphism in the pathogenesis of malignant disorders. In a Chinese population, the susceptibility of gastric cancer was higher in patients carrying an A allele [19]. Breast cancer was found to be more common in the AG heterozygous group [20]. On the other hand, no predisposing role of any *CTNNB1* (rs4135385) polymorphism could be confirmed in terms of multiple myeloma. However, cyclophosphamide – thalidomide – dexamethasone (CTD) combination therapy was more effective in MM patients carrying the AA genotype [11]. Our results were in accordance with this finding as therapeutic responses and progression-free survival results were more favourable in AA homozygous patients after Imid-based therapies. Moreover, *CTNNB1* (rs4135385) polymorphism were found to be an independent prognostic factor in terms of PFS results besides ISS stages and FISH results. No associations were detected between beta-catenin gene polymorphisms and the severity of any Imid-related side effects. Being aware of this polymorphism, personalized treatment strategies can be suggested for multiple myeloma patients.

Glutathione-S-transferase enzyme plays an important role in the metabolism of carcinogens and cytostatic agents as well. *GSTP1* 105 variant of this enzyme has less ability for detoxification, therefore the effectivity of cytostatic agents is increased in such multiple myeloma patients. In patients carrying the GSTP1 105Ile homozygous genotype, event-free and overall survival results were reported to be more favourable after alkylating agent or anthracyclin-based treatment [16]. In our cohort, neither *GSTP1* 105, nor *GSTP1* 114 polymorphisms had any impact on the treatment results and survival data.

Our results highlighted that beta-catenin gene rs4135385 gene polymorphism may influence the clinical features of multiple myeloma patients and have some impact on the treatment results and survival data after Imid-based therapies. Determination of molecular patterns and gene polymorphisms in multiple myeloma patients is an important aspect of personalized medicine which may improve patients’ survival. [2].
